# Prodrugs of pyrophosphates and bisphosphonates: disguising phosphorus oxyanions

**DOI:** 10.1039/d1md00297j

**Published:** 2022-03-01

**Authors:** Emma S. Rudge, Alex H. Y. Chan, Finian J. Leeper

**Affiliations:** Yusuf Hamied Department of Chemistry, University of Cambridge Lensfield Road Cambridge CB2 1EW UK fjl1@cam.ac.uk

## Abstract

Pyrophosphates have important functions in living systems and thus pyrophosphate-containing molecules and their more stable bisphosphonate analogues have the potential to be used as drugs for treating many diseases including cancer and viral infections. Both pyrophosphates and bisphosphonates are polyanionic at physiological pH and, whilst this is essential for their biological activity, it also limits their use as therapeutic agents. In particular, the high negative charge density of these compounds prohibits cell entry other than by endocytosis, prevents transcellular oral absorption and causes sequestration to bone. Therefore, prodrug strategies have been developed to temporarily disguise the charges of these compounds. This review examines the various systems that have been used to mask the phosphorus-containing moieties of pyrophosphates and bisphosphonates and also illustrates the utility of such prodrugs.

## Abstract 2

Pyrophosphates (diphosphates) and bisphosphonates are potentially important drugs but are anionic and too polar to penetrate cells; here we review approaches that have been taken to create cell-permeable prodrugs of these type of compounds.

## Graphical abstract

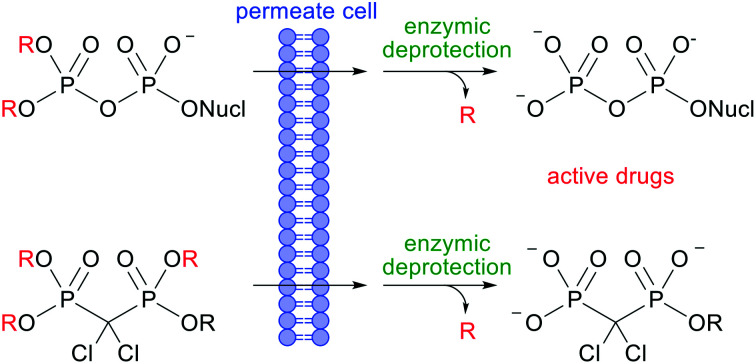

## Introduction

**Fig. 1:**
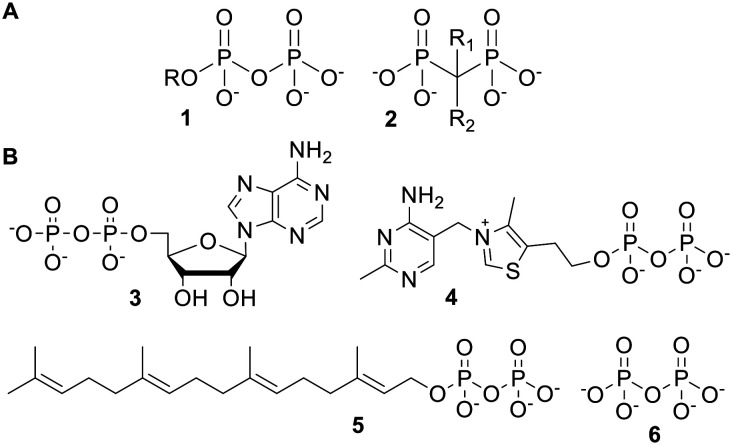
(A) General structures of pyrophosphates and bisphosphonates; (B) some biologically relevant pyrophosphates.

Pyrophosphates (or diphosphates) 1 and bisphosphonates 2 (Fig. 1A) are broad classes of organic compounds, which contain geminal phosphorus atoms. Pyrophosphates contain a P–O–P backbone, whereas bisphosphonates have a P–C–P linkage which is much more resistant to hydrolysis.^1^ Bisphosphonates have thus been used as pyrophosphate analogues with greater stability in aqueous media.^2,3^

Pyrophosphates include many naturally occurring molecules with important biological activities. Selected examples are shown in Fig. 1B. Adenosine diphosphate (ADP) 3 is interchanged with adenosine triphosphate (ATP), which is the ‘universal energy currency’ of cells.^4^ Thiamine pyrophosphate (TPP) 4 is a cofactor used by multiple enzymes, including those involved in carbohydrate and branched amino acid metabolism.^5^ Geranylgeranyl pyrophosphate (GGPP) 5 and other pyrophosphates are biosynthetic precursors to terpenes.^6^ Finally, inorganic pyrophosphate (PP_i_) 6 is used to regulate physiological processes such as calcification.^3^

It can be useful to introduce exogenously derived pyrophosphates into living systems in order to study their biological effects. Analogues of these molecules (including bisphosphonates) can be used similarly, or else they may be intended to disrupt or amplify a particular cellular process (*e.g.* as a drug). The main barrier to using pyrophosphates in this way is their high negative charge, which precludes passive diffusion through the lipid bilayers that constitute biological membranes.^7^ This causes slow cell uptake and poor bioavailability. To illustrate, the p*K*_a_ values of PP_i_ are 1.52, 2.36, 6.60 and 9.25.^8^ Thus pyrophosphates can exist as trianions at physiological pH. Bisphosphonates have a similarly high negative charge density: the p*K*_a_ values of methylene bisphosphonate (MBP, 2 R_1_ = R_2_ = H) are <2, 2.57, 6.87 and 10.33.^9^ Another issue with using pyrophosphates as drugs is that they can be rapidly hydrolysed by enzymes such as phosphatases, *e.g.* in human serum.^1^

Prodrug strategies can be used both to increase the bioavailability and cell uptake of charged molecules and to provide resistance to degradation in systemic circulation.^10,11^ A prodrug is a derivative of a bioactive compound which can be converted to the parent compound in living systems by chemical or enzyme-catalysed reactions.^12,13^ In the case of monophosph(on)ate prodrugs strategies are well developed and there are four nucleotide analogue prodrugs already in clinical use (see below). This review describes pyrophosphate and bisphosphonate prodrugs that have been explored, considering only systems in which the phosphorus-containing moiety is disguised. Although there have been two reviews by Meier on his group's strategy for pyrophosphate prodrugs,^14,15^ and one by Vepsäläinen explaining his group's work on bisphosphonate prodrugs,^16^ a comprehensive review such as is provided here is absent in the literature.

## Pyrophosphate prodrugs

Prodrugs of pyrophosphates have been almost exclusively used in the context of nucleoside analogues. Nucleoside analogues are themselves prodrugs, as they undergo stepwise phosphorylation catalysed by intracellular kinases to eventually yield the nucleoside triphosphate (NTP).^17^ This metabolite can inhibit the action of nucleic acid polymerases, thereby giving rise to the anticancer and antiviral activity of this class of compounds.^18^ The main issue with this strategy is that the activating enzymes often have high substrate specificities, which can diminish or can even abolish the therapeutic effect of the nucleoside analogue.^19^ Furthermore, downregulation of these enzymes can lead to the emergence of resistant strains. Therefore, there is a need to achieve ‘kinase bypass’ by introducing nucleotides directly.^20^ However, phosphorylated species are polyanionic at physiological pH so ‘pro-nucleotides’ (nucleotide prodrugs) are required for efficient cell entry.^21^

Often the first phosphorylation to produce the nucleoside monophosphate (NMP) is rate limiting, in which case an NMP prodrug may have better activity than the parent nucleoside. Indeed, a plethora of strategies for masking NMPs have been developed.^22–26^ However, in other cases the ‘bottleneck’ in the activation of a nucleoside analogue is the second phosphorylation step, which produces the nucleoside diphosphate (NDP) from the NMP. This is the case for 3′-azido-3′-deoxythymidine (AZT), a drug of the nucleoside analogue reverse transcriptase inhibitor (NRTI) class which is used to treat HIV.^27^ Not only does this limit the therapeutic benefits of the drug but also the resulting intracellular accumulation of AZTMP can cause severe side effects.^28^ An NDP prodrug would therefore possess a clear advantage.

The NMP kinases that effect the second phosphorylation are generally substrate-selective, with thymidylate kinase, uridylate–cytidylate kinase, and several adenylate kinases and guanylate kinases known in humans.^17^ So, analogues of NMPs may well be poor substrates. We are not aware of any other nucleoside analogues, other than AZT and nucleosides from 22a and b (see later, Fig. 7), where it has been shown that the second phosphorylation of the analogue is rate-limiting, but only a few nucleoside analogues have been studied to this level of detail, so there are likely to be others.

Surprisingly few groups have attempted to design NDP prodrugs. This can be attributed to unfavourable properties of the phosphoric anhydride bond. This bond is thermodynamically unstable (in fact hydrolysis of this ‘high-energy’ bond in ATP provides energy to drive cellular processes). However, the phosphoric anhydride bond is kinetically stable due to the high negative charge density at the phosphate oxygens, which both repels nucleophiles and ensures each phosphate-containing moiety is a poor leaving group^7^ (ATP-dependent enzymes contain cations such as Mg^2+^ in their active sites to complex the phosphates and thereby increase the rate of hydrolysis). This presents a paradox in the design of pyrophosphate prodrugs, as to increase the rate of transmembrane diffusion the anionic character of the compound must be reduced, but completely masking each negative charge would render the phosphoric anhydride bond extremely unstable. Prodrug strategies for NDPs have therefore relied on maintaining a negative charge at the α-phosphate, whilst appending suitably lipophilic promoieties at the β-phosphate to enable membrane penetration.

### Acyl promoieties

**Fig. 2:**
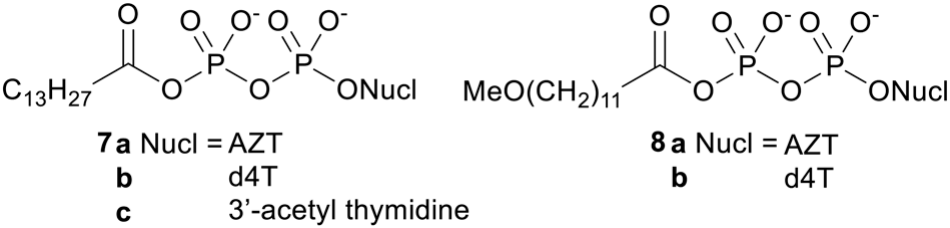
Acyl NDP prodrugs studied by Huynh-Dinh and coworkers.^29,31^

The first attempt to produce an NDP prodrug was reported by Huynh-Dinh and coworkers in 1995.^29^ This group noted that the carboxylic–phosphoric anhydride bond in acetyl phosphate is hydrolysed more readily than the pyrophosphate bond in ATP. They therefore reasoned that in acyl pyrophosphates (*e.g.*7 and 8, Fig. 2) hydrolysis should selectively occur at the carboxyl group, leaving the phosphoric anhydride bond intact. They also hypothesised that they would be able to modulate the physicochemical properties of the prodrug by varying the fatty acid-derived acyl group attached to the terminal phosphate.^30^

Hydrolysis studies of the prodrugs were carried out in pH 7.0 triethylammonium acetate (TEAA) buffer and in Roswell Park Memorial Institute (RPMI) cell culture medium.^31^ In both media the anhydrides were cleanly hydrolysed to the desired NDP. However, their half-life in RPMI medium was much shorter due to the higher concentration of nucleophiles such as amino acids, proteins and inorganic salts in this medium (for example the half-life of 7a was 114 h in TEAA and 1.7 h in RPMI medium). Accordingly, when tested *in vitro* there was no difference between the antiretroviral activity of the prodrugs and that of their parent nucleosides. This indicates that the prodrugs were not able to increase the rate of cell uptake and instead were hydrolysed to the parent compounds in the cell culture medium.

A pyrophosphate prodrug with better cell-penetrating ability could potentially be produced by protecting two or three of the anionic oxygen atoms with acyl groups. However, a search of the literature did not reveal any instances in which this has been tried.

### Salicyl alcohol promoiety

**Fig. 3:**
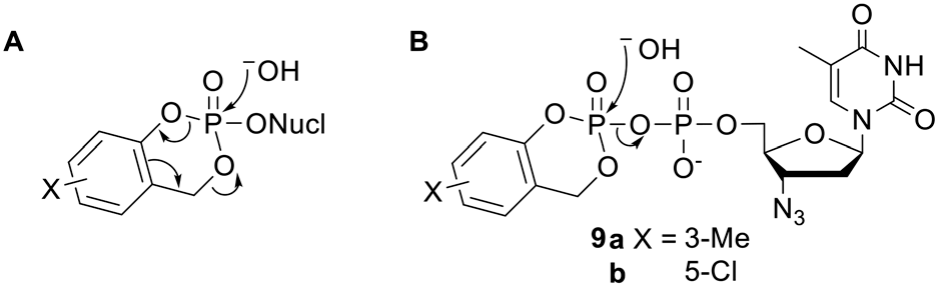
(A) Hydrolysis pathway of *cyclo*Sal NMP prodrugs. (B) Major hydrolysis pathway of *cyclo*Sal AZTDP prodrugs.

The ‘*cyclo*Sal’ approach is a prodrug strategy that was developed by Meier and coworkers, for the protection of NMPs.^22,32^ Recognising the advantages of prodrugs of higher phosphorylated species, the group applied the same system to NDPs by diesterifying the β-phosphorus with a salicyl alcohol progroup.^33,34^ Note the use of just one promoiety to mask two negative charges – this strategy can be used to limit the molecular weight of prodrugs. *Cyclo*Sal NMPs are deprotected by chemical hydrolysis (Fig. 3A). Importantly, the initial activating step involves nucleophilic attack on the phosphorus atom. However, the equivalent attack on *cyclo*Sal-AZTDP 9a (Fig. 3B) led almost entirely to hydrolysis of the phosphoric anhydride bond (despite this generating a leaving group in which a single phosphate moiety bears two negative charges). Thus the major product released was the NMP (99%) rather than the NDP (1%).^15^ Meier and coworkers tried to accelerate phenyl-phosphate ester hydrolysis by changing the benzene ring substituent to a more electron-withdrawing chlorine atom (9b) but unproductive phosphoric anhydride cleavage was still favoured (75% NMP *vs.* 25% NDP). The group therefore surmised that the only feasible way to produce an NDP prodrug would be to use a promoiety that could be removed without any sort of nucleophilic attack at a phosphoryl group.

### Acyloxybenzyl promoieties

Esterification with acyloxybenzyl groups is another prodrug strategy that has been used in the case of NMPs.^35^ Unlike the carboxylic acid and *cyclo*Sal promoieties described above, acyloxybenzyl groups are removed in biological media by an enzymatically triggered process. This type of unmasking is often considered to be more useful than activation which relies solely on chemical hydrolysis because the bioactive compound will only be released in environments that contain the activating enzymes.^13^ For example, this can ensure that a prodrug remains intact until it reaches the systemic circulation or has penetrated a specific cell type or intracellular compartment.^19^ This can lead to a high drug concentration in the desired location as once the highly polar drug has been unmasked it will no longer be able to freely diffuse across membranes.^11^ In addition, better targeting to particular locations can make side effects less likely. Often prodrugs are designed to be activated by lipases or other esterases as these enzymes are present at high intracellular concentrations.^36^

**Fig. 4:**
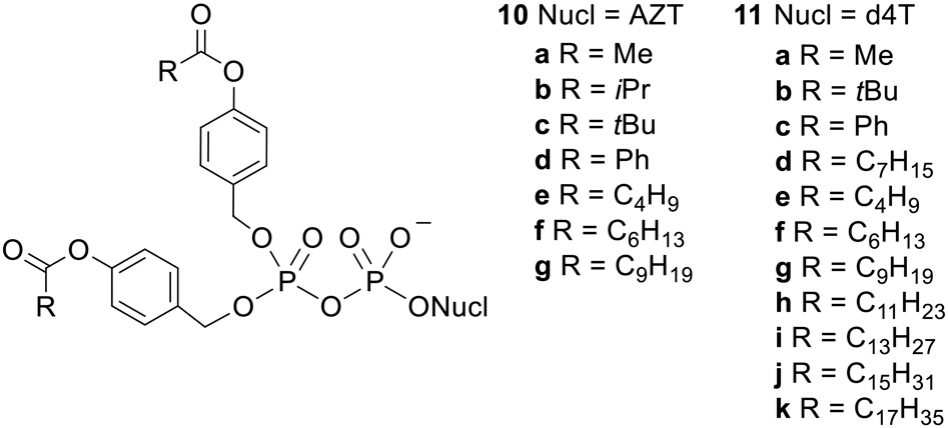
Bis(acyloxybenzyl)NDP prodrugs studied by Meier and coworkers.^26,33,34,37^

Crucially, the initial step in the activation of acyloxybenzyl-masked prodrugs is (carboxyl)esterase-mediated hydrolysis of the acyl ester, which is separated from the phosphoryl moiety by a benzyl linker, *i.e.* nucleophilic attack occurs some distance away from the phosphorus centre. Meier and coworkers therefore synthesised NDP prodrugs 10 and 11 (Fig. 4) in which the β-phosphate is esterified with two 4-acyloxybenzyl groups and the α-phosphate is left unprotected.^26,33,34,37^ As noted above, it is important that some negative charge is maintained at the phosphoric anhydride bond or else hydrolysis would be extremely facile in aqueous solutions. This design also allows the physicochemical properties of the compound to be modulated by changing the acyl group and/or the substituents on the benzene ring.

**Scheme 1:**
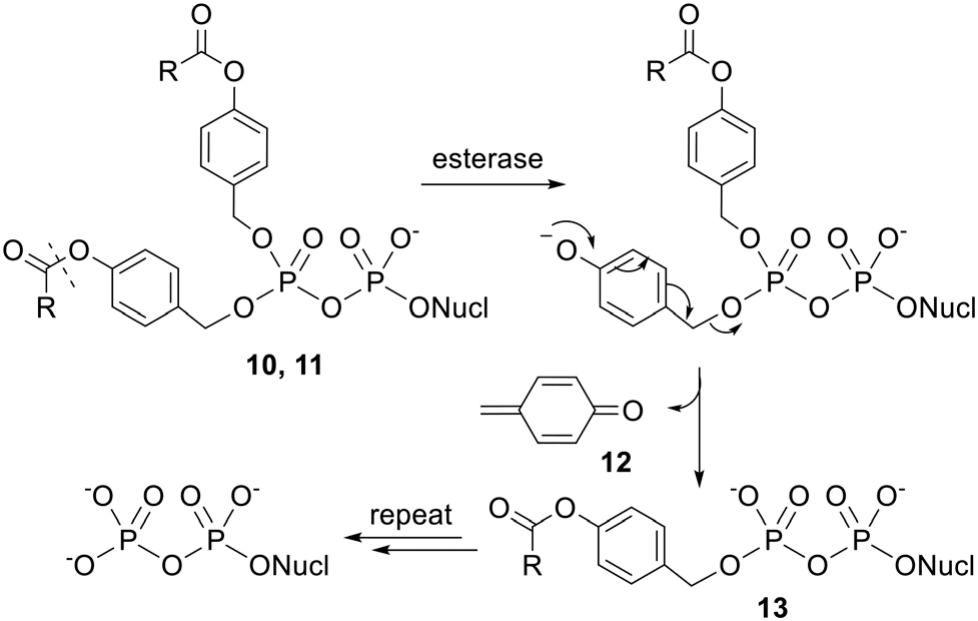
Mechanism of activation of Di*PP*ro-nucleotides.

The mechanism of activation of these bis(4-acyloxybenzyl) NDP prodrugs (which have been coined ‘Di*PP*ro-nucleotides’) is shown in Scheme 1. The initial acyl ester hydrolysis converts the ester substituent into a much more electron-donating hydroxyl substituent. This change in polarity results in a spontaneous fragmentation in which a singly masked intermediate 13 is expelled. Repetition of this process cleaves the second benzyl-phosphate bond and releases the bioactive form of the NDP. Note that a by-product of these reactions is a cyclic dienone 12. This will be susceptible to attack by nucleophiles and is therefore a potential source of toxicity.

Initial studies showed that compounds 10a–d and 11a–d were relatively stable in aqueous buffer solution whilst 10a and 11a were rapidly degraded in cell extracts to yield predominantly the NDP.^34^

For instance, bis(acetoxybenzyl)-AZTDP 10a had a half-life of 17 h in pH 7.3 phosphate-buffered saline (PBS), but had an 500-fold shorter half-life (2 min) in T lymphocyte (CEM/0) cell extracts. These half-lives refer to the first deprotection; the second deprotection is somewhat slower, but only marginally so in cell extracts (*t*_1/2_ = 3 min), and the final product was >95% AZTDP. The authors state that similar high stability was observed in citrate/HCl buffer at pH 2.0. In 20% human plasma and in RPMI media with 10% heat-inactivated fetal calf serum the prodrugs were more stable than in cell extracts but less stable than in PBS. As expected for steric reasons, the esters containing branched alkyl groups were hydrolysed more slowly in all media tested. It was also noticed that in biological media phosphoric anhydride bond cleavage could compete with acyl ester hydrolysis if the initial deprotection step was slow. Once the first masking group had been removed however, the extra negative charge present in intermediate 13 protected the phosphoric anhydride bond from hydrolysis. The authors concluded that half-lives of 1–30 min were required for selective formation of the NDP over the NMP.

Di*PP*ro-nucleotides 11a–d were then tested for their ability to inhibit HIV replication in T lymphocytes.^34^ All the compounds evaluated had similar or slightly worse activity than the (unphosphorylated) parent nucleoside 2′,3′-dideoxy-2′,3′-didehydrothymidine (d4T) in wild-type (CEM/0) cells. This result alone does not prove the ability of the Di*PP*ro compounds to deliver phosphorylated species inside cells because the prodrugs could have been hydrolysed (at the acyl ester or the phosphoric anhydride bond) in the extracellular medium and subsequently dephosphorylated to yield the active parent compound. More importantly, in a thymidine kinase-deficient (CEM/TK^−^) cell line prodrugs 11c and 11d were 82- and 27-fold more effective than d4T (EC_50_ values were 0.85 μM for 11c, 2.6 μM for 11d and 70 μM for d4T). Thymidine kinase (TK) is the enzyme responsible for monophosphorylating thymine-containing nucleosides. Thus d4T is very poorly converted to d4TTP in the mutant cell line, which abrogates its activity. The fact that prodrugs 11c and 11d were still active in this cell line proves that they were able to enter the cells and release phosphorylated compounds intracellularly. It does not prove they delivered the NDP (as opposed to the NMP) into cells, although the hydrolysis studies in cell extracts suggest they did. The enzyme which converts the thymidine monophosphate into the diphosphate is thymidylate kinase (TMPK) but cell lines lacking this enzyme do not exist since it is essential for cell viability.

Meier and coworkers suggest that compound 11a failed to bypass TK as effectively as 11c and 11d because it was still too polar for efficient transmembrane diffusion. Therefore, to investigate the relationship between lipophilicity and biological activity they synthesised Di*PP*ro-nucleotides 11e–k, which contained fatty acids with longer alkyl chains.^37^ As expected based on steric hindrance, they found these prodrugs were hydrolysed more slowly in PBS and CEM cell extracts. Concomitant with this increase in stability was a decrease in the ratio of NDP : NMP formed, which fell to 1.5 : 1 for the largest molecules. In the *in vitro* anti-HIV test the prodrugs were generally equally or slightly less active compared to the parent nucleoside. The most potent compound was the *C9*-Di*PP*ro-nucleotide 11g, which exhibited an EC_50_ value an order of magnitude lower than that of d4T in CEM/0 cells. In addition, only prodrugs with R = C_6_H_13_ or longer fully retained their activity in the CEM/TK^−^ assay, which suggests these compounds were lipophilic enough for efficacious transmembrane passage.

**Fig. 5:**
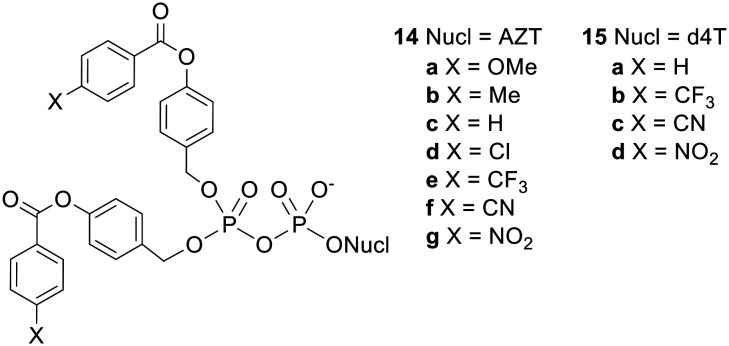
Bis(benzoyloxybenzyl) NDP prodrugs studied by Meier and coworkers.^38^

Although the fatty acid-containing Di*PP*ro-nucleotides were adequately lipophilic, their increased stability to acyl ester hydrolysis allowed phosphoric anhydride bond cleavage to compete, leading to the formation of increased amounts of NMP. Seeking to find prodrugs that were sufficiently hydrophobic but also hydrolysed fast enough in cell extracts to allow selective NDP release, Meier's group synthesised a series of bis(benzoyloxybenzyl) NDP prodrugs 14 and 15 (Fig. 5) with various substituents at the 4-position of the benzoyl moiety.^38^ They found that as the substituent became more electron withdrawing, the rate of promoiety hydrolysis increased. However, these electron-acceptor-substituted compounds did not fully retain their inhibitory activity in CEM/TK^−^ cells, possibly because they were partially hydrolysed in the cell culture medium prior to cell uptake.

**Fig. 6:**
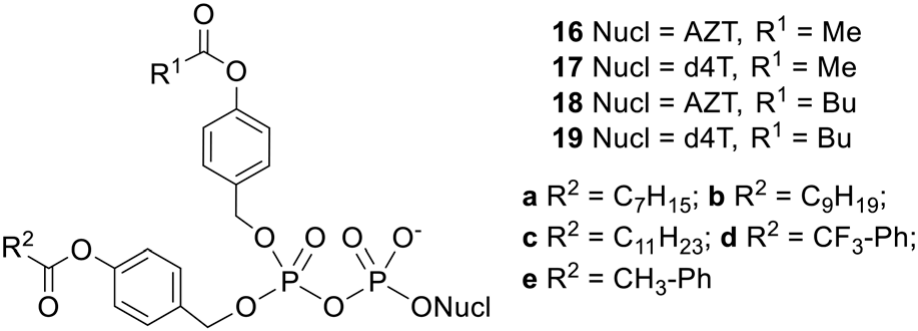
Non-symmetric bis(acyloxybenzyl) NDP prodrugs studied by Meier and coworkers.^26^

NDP prodrugs that could efficiently penetrate cells and selectively release the NDP were finally achieved with the design of a second generation of di*PP*ro-nucleotides 16–19 (Fig. 6), which comprised two different acyloxybenzyl masking units.^26^ It should be noted that these compounds contain a chiral centre at the β-phosphorus and thus exist as two diastereomers which may have different biological activities. In these non-symmetric prodrugs one progroup contains a long alkyl chain and is intended to confer hydrophobicity to the molecule whilst the other contains a short alkyl chain and is intended to be hydrolysed rapidly by intracellular esterases so as to avoid phosphoric anhydride cleavage. Selective NDP release was observed in CEM cell extracts: for example, whereas the NDP : NMP ratio produced by the symmetric *C9C9*-prodrug 10g was 1.5 : 1, the ratio from the non-symmetric *C1C9*-di*PP*ro compound 16b was 5 : 1. The small fraction of NMP formed was likely due to dephosphorylation of the released NDP by phosphatases in the biological medium rather than hydrolysis of the prodrug phosphoric anhydride bond, since incubation of AZTDP in the cell extracts generated an NDP : NMP ratio of 3 : 1. The non-symmetric prodrugs were all active against HIV in CEM/0 cells, with some even being more potent than the parent nucleosides. Moreover the activities improved as the length of the alkyl chain in the lipophilic masking unit was increased. Whilst compounds 19b and 19c maintained their activity in CEM/TK^−^ cells, the other prodrugs were less potent in the mutant cell line, with the benzoyl-containing d4TDP prodrugs and each of the AZTDP prodrugs losing more or less all antiviral activity. For the most part, this can be explained in terms of insufficient hydrophobicity, but the different behaviours of the d4T and AZT prodrugs containing identical masking units is more puzzling. Possibly, the delivery of large amounts of AZTDP into cells by the prodrugs inhibited its conversion to AZTTP.^39^

**Fig. 7:**
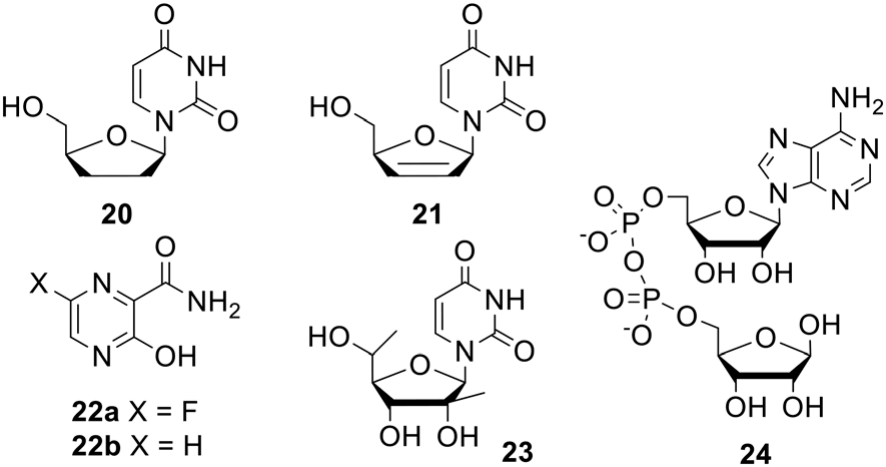
Compounds to which the di*PP*ro concept has been applied.

Since its invention the di*PP*ro concept has been applied to other nucleoside analogues and related compounds (Fig. 7). 2′,3′-Dideoxyuridine (ddU) 20 and 2′,3′-dideoxy-2′,3′-didehydrouridine (d4U) 21 are two nucleoside analogues which completely lack *in vitro* activity despite their triphosphate metabolites being highly effective inhibitors of HIV reverse transcriptase.^40–42^ Meier and coworkers synthesised symmetrical bis(acyloxybenzyl) prodrugs of ddU and d4U in an effort to overcome their inefficient intracellular activation but it transpired that the conversion of the NDPs to NTPs was then rate limiting.^43^ In another paper the same group found that NDP formation was a significant bottleneck in the activation of nucleobase analogues T-705 22a and T-1105 22b.^44^ Both symmetric and non-symmetric di*PP*ro NDPs had more potent anti-influenza virus activity than the parent pseudobases in wild-type MDCK cells. In addition the prodrugs retained their activity in an MDCK-TG^res^ cell line that was incapable of activating the parent compounds, proving their ability to deliver nucleotides into these cells. Accordingly, the *C9C9*-derivative was found to suppress influenza viral RNA synthesis at lower concentrations than the parent nucleobase. In a third nucleotide application, *C9C9*-acyloxybenzyl prodrugs of 5′-*R* and 5′-*S* 2′,5′-dimethyluridine 23 were used by Dasari *et al.* to test whether the second phosphorylation is the rate-limiting step in the activation of the nucleoside analogue.^45^ Lastly Pahnke and Meier used a 4-pentanoyloxybenzyl group to mask adenosine diphosphate ribose (ADPR) 24, a potent activator of the TRPM2 ion channel.^46^ This protection was shown to be reversible in a solution containing pig liver esterase.

**Fig. 8:**
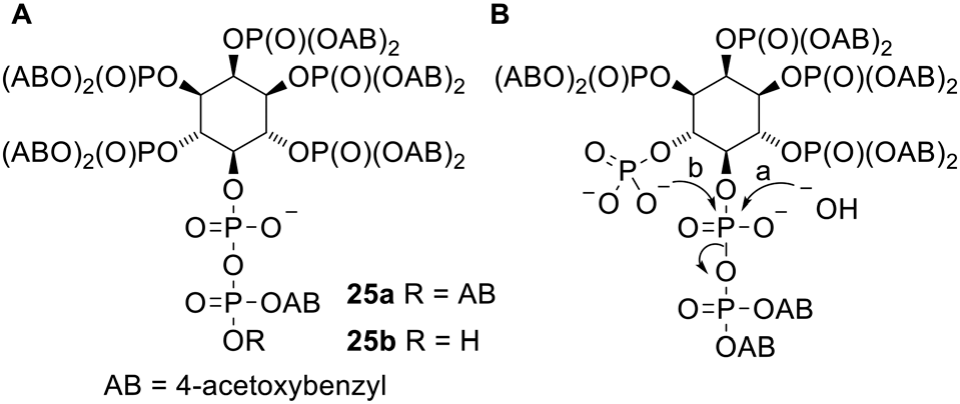
(A) Acetoxybenzyl 5-PP-*myo*-InsP_5_ prodrugs studied by Jessen and coworkers;^48^ (B) undesired degradation pathways of (partially unmasked) prodrug 25a.

The di*PP*ro strategy has also been used in one non-nucleotide context. Diphospho-*myo*-inositol phosphates (*x*-PP-InsP_*y*_, where *x* indicates the number of the atom of the inositol bearing the pyrophosphate group and *y* indicates the number of monophosphate groups attached) are a family of intracellular secondary messengers with important roles in cell signalling.^47^ However it is difficult to study the functions of these compounds as they are extremely polar and therefore cannot enter cells through passive diffusion. To solve this problem Jessen and coworkers synthesised a prodrug version of 5-PP-*myo*-InsP_5_ (Fig. 8A) in which all 5 monophosphates and the β-phosphate of the pyrophosphate are masked with 4-acetoxybenzyl (AB) groups.^48^ [*S*-Acetylthioethyl (SATE) promoieties were also investigated but were removed extremely slowly in biological media.] This (AB)_12_ compound 25a displayed poor aqueous solubility but nevertheless was shown to release the parent metabolite in mammalian tissues, cell homogenates and *Dictyostelium discoideum* extracts. Two side products were also observed: the first was determined to be InsP_6_ (the compound formed by pyrophosphate hydrolysis, pathway a in Fig. 8B). This was formed even in the presence of NaF, which inhibits diphosphoinositol phosphatases, suggesting that the phosphoric anhydride bond in the prodrug was susceptible to hydrolysis despite the α-phosphate being left negatively charged. The second by-product was suggested to be a cyclic anhydro version of InsP_6_, which was probably formed by nucleophilic attack of an unmasked monophosphate onto the pyrophosphate α-phosphate (pathway b in Fig. 8B) since the doubly protected β-phosphate is a reasonable leaving group. In contrast the amounts of these by-products formed from an (AB)_11_ compound 25b (in which the pyrophosphate has only one masking unit) were significantly reduced due to the extra negative charge on the terminal phosphate. When tested *in vitro* compound 25a was able to enter HCT116 cells and showed ‘robust’ intracellular release of 5-PP-InsP_5_. Compound 25b was also able to deliver 5-PP-InsP_5_ inside cells, although cell uptake was reduced because of the extra negative charge.

## Bisphosphonate prodrugs

As mentioned above, a major drawback of using pyrophosphates as drugs is that they can be rapidly dephosphorylated by enzymes such as phosphatases. To get around this the bridging oxygen atom can be substituted for a carbon atom, *i.e.* a bisphosphonate analogue can be used instead. However it must be noted that due to the electronegativity difference between carbon and oxygen, this modification could also affect other properties of the molecule such as its ionisation state and/or affinity for an enzyme or receptor.^1^

**Fig. 9:**
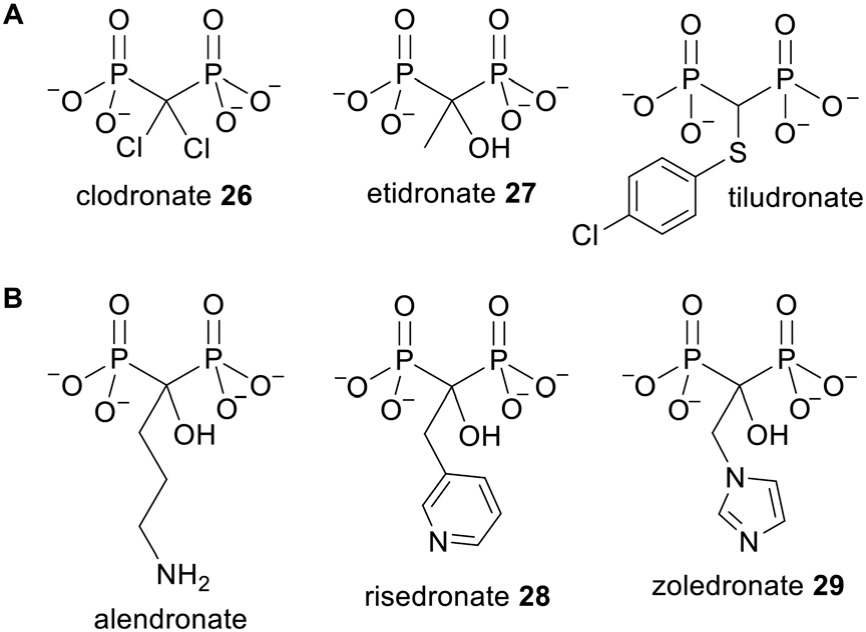
Examples of (A) non-nitrogenous bisphosphonates and (B) nitrogenous bisphosphonates.

Bisphosphonates were originally synthesised in the 19th century^49^ and were first approved for clinical use in 1977.^50^ Representative examples of clinically used bisphosphonates are shown in Fig. 9. Bisphosphonates are primarily used as bone resorption inhibitors for the treatment of diseases including osteoporosis, Paget's disease and bone metastasis.^51^ Owing to their high negative charge they bind strongly to divalent cations including Ca^2+^, which is found in large quantities in bone mineral, hydroxyapatite.^52^ Bisphosphonates therefore adsorb to the surface of bones, where they can be taken up by osteoclasts (bone-destroying cells) *via* fluid-phase endocytosis.^53^ There are two main categories of bisphosphonates: nitrogenous bisphosphonates (NBPs) and non-nitrogenous bisphosphonates, and these have different intracellular mechanisms of action.^54,55^ The latter class are analogues of PP_i_ and thus undergo a reaction with aminoacyl adenylates catalysed by aminoacyl-tRNA synthetases.^56^ This yields β,γ-methylene analogues of ATP which can inhibit the mitochondrial ATP–ADP translocase, thereby inducing cell cycle arrest and/or apoptosis.^57,58^ On the other hand the nitrogen-containing bisphosphonates act by inhibiting farnesyl pyrophosphate synthase (FPPS), a cytosolic enzyme involved in the mevalonate pathway of isoprenoid biosynthesis.^59^ This depletes cells of intermediates such as farnesyl pyrophosphate (FPP) and geranylgeranyl pyrophosphate (GGPP), which are substrates for transferase enzymes that prenylate small G proteins such as Ras, Rac, Rab, Rap and Rho. These GTPases have crucial roles in signal transduction and require prenylation for plasma-membrane localisation. Without this, cellular processes such as proliferation, survival and migration are inhibited and eventually apoptosis is induced. Inhibition of FPPS and/or GPPS also causes upstream isoprenoid precursors such as isopentenyl pyrophosphate (IPP) and dimethylallyl pyrophosphate (DMAPP) to accumulate inside cells. IPP can then be metabolised to ApppI, another cytotoxic ATP analogue.^60–62^

Bisphosphonates are trianionic under physiological conditions, which causes poor gastrointestinal absorption when they are orally dosed.^63^ Drug molecules can enter the systemic circulation from the intestinal lumen through either a transcellular or a paracellular pathway. The transcellular route requires the molecule to be lipophilic enough for passive diffusion across first the apical membrane and then the basolateral membrane of the gut epithelial cells. As an illustration the log *P* of clodronate 26 (Fig. 9) will be less than −5.4 (the log *P* of the monoethyl ester of clodronate^64^), thus it is not membrane permeable. Bisphosphonates instead utilise the paracellular pathway, which involves passage through the spaces between cells.^65^ However this is generally much less efficient than transcellular transport because the gateways are narrow and tight junctions serve to block the entry of any molecule with a molecular weight greater than 150.^63^ Another problem is that bisphosphonates can form poorly soluble complexes with divalent cations in the intestinal lumen (such as Ca^2+^ and Fe^2+^ ions from food), which further hinders their uptake.^66,67^ The oral bioavailability of bisphosphonates thus tends to be extremely low (for instance for clodronate it is only 1–2%).^68^ Consequently high oral doses are required and this can lead to severe irritation of the digestive tract.^66^ Bisphosphonate prodrugs have been sought to alleviate these issues.^67^

More recently bisphosphonates have been found to have anticancer properties, even in extraskeletal environments.^69,70^*In vitro* experiments have shown that NBPs can inhibit tumour cell proliferation, migration and survival in a variety of cell lines.^71^ Moreover in a clinical setting, use of imidazole-containing NBP zoledronate 29 (Fig. 9) in addition to standard therapies led to an increase in disease-free survival in breast cancer patients and an increase in overall survival in multiple myeloma and lung cancer patients.^72–75^ However the rapid localisation of bisphosphonates to bone and their slow internalisation by cells that are not highly pinocytotic limits their usefulness for treating soft-tissue tumours. A prodrug strategy thus has the potential to broaden the therapeutic scope of these drugs.

Bisphosphonates have additionally been shown to stimulate the proliferation of human γδ T cells expressing Vγ2Vδ2 T-cell receptors (TCRs), also termed Vγ9Vδ2 TCRs, and may aid immunotherapy cancer treatments.^76,77^ At the same time, bisphosphonates can sensitise tumour cells towards the cytotoxic activity of γδ T cells.^78^ These effects are due to FPPS inhibition, which causes IPP and DMAPP to accumulate inside cells and bind to the intracellular domain of the receptor butyrophilin (BTN) 3A1.^79–81^ This interaction is recognised at the cell-surface by γδ T cells through their TCRs and induces their cell-killing activity.^82^ Successful clinical trials of adoptive transfer of γδ T cells for treatment of cancer have used zoledronate as an adjuvant.^83,84^ However, *in vitro* studies have shown that a bisphosphonate prodrug, tetrakis-pivaloyloxymethyl 2-(thiazole-2-ylamino)ethylidene-1,1-bisphosphonate (PTA) 49 (Fig. 16), is much more potent than zoledronate in stimulating Vγ2Vδ2 T cells to proliferate, lyse tumour cells and secrete tumour-necrosis factor alpha (TNF-α).^85^ These improvements in activity can be attributed to the prodrug being suitably lipophilic for efficient cell uptake.

### Alkyl promoieties

**Fig. 10:**
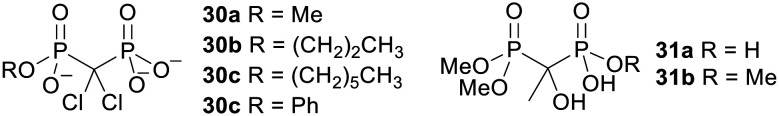
Alkyl esters of clodronate and etidronate studied by Vepsäläinen and coworkers.^88,89^

Arguably, one of the simplest ways to increase the hydrophobicity of bisphosphonates is to mask some or all of the phosphonate oxygen atoms with alkyl groups.^64,86^ It was originally hypothesised that the bisphosphonate esters thus derived would be susceptible to hydrolysis by intracellular enzymes such as phosphodiesterases and phosphatases. Many different alkyl esters of bisphosphonates have been synthesised.^87*a*–*g*^ However, when the hydrolysis kinetics of clodronate monoesters 30 (Fig. 10) were studied by Vepsäläinen and coworkers, none of the compounds were degraded during 4 h in 80% human serum or rabbit liver homogenate and methyl ester 30a was also stable during 28 days in pH 7.4 phosphate buffer.^88^ Etidronate diester 31a and triester 31b were not hydrolysed in these media either.^89^ Hence these derivatives are analogues of bisphosphonates as opposed to prodrugs.

### Aryl promoieties

**Fig. 11:**
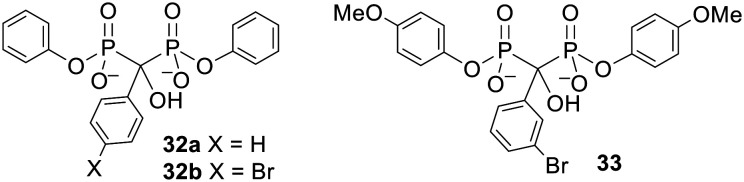
Aryl esters studied by Lecouvey and coworkers.^93,94^

Bisphosphonate esters containing aryl protecting groups have also been synthesised.^90–92^ In contrast to the alkyl-masked bisphosphonates, there is some evidence that these compounds can act as prodrugs. This can be rationalised by noting that phenols are generally better leaving groups than alkyl alcohols. For instance, bisphosphonates protected by a single phenyl group at each phosphonate moiety 32 (Fig. 11) were found by Lecouvey and coworkers to inhibit HuH7 hepatocarcinoma cell viability more potently than their tetraacid forms.^93^ Adding 3-isobutyl-1-methylxanthine (IBMX, a phosphodiesterase inhibitor) to the assay reduced the activity of the diesters but not the acids. This suggests that a) bisphosphonate esterification enhanced cell entry, b) intracellular deprotection is necessary for the compounds to show anticancer activity and c) phosphodiesterases are responsible for hydrolysis of the bisphosphonate esters. Five-day hydrolysis studies showed that compound 32b remained intact in water, cell culture medium and human serum and confirmed it could be degraded to the parent bisphosphonate in cell extracts. Interestingly Vepsäläinen and coworkers had found that a monophenyl ester of clodronate 30d was not susceptible to hydrolysis during 4 h in 80% human serum or during 6 h in 10% rabbit liver homogenate.^88^ The results with 32 suggest that this time period is not long enough to determine whether or not phenyl esters of clodronate can be used as prodrugs.

Another bisphosphonate was symmetrically diesterified with 4-methoxyphenyl groups to produce prodrug 33 (Fig. 11).^94^33 was less hydrophilic than the parent bisphosphonate (log *P* values were −0.75 for the parent and −0.31 for 33) and correspondingly was found to have a more powerful effect on breast cancer cell proliferation, survival and migration. 33 was also better at reducing angiogenesis in nude mice with breast cancer cell xenografts and (unlike its parent compound) could inhibit metastasis. Hence this work provides a proof of concept that phenyl esters can be used as prodrugs to improve the anticancer properties of bisphosphonates *in vivo*.

### Acetal promoieties

**Fig. 12:**
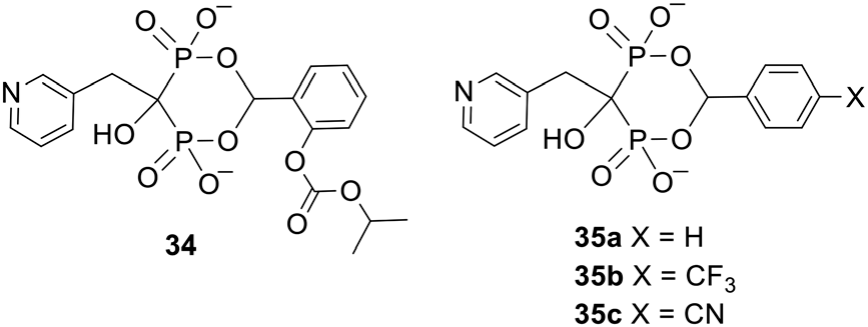
Cyclic esters of risedronate studied by Pavlov *et al.*^96^

Another type of bisphosphonate ester comprises a cyclic structure in which a single progroup is used to mask both phosphonates.^95^ Again, this helps to reduce the molecular weight of the prodrug. Pavlov *et al.* applied this system to risedronate 28 to produce benzaldehyde derivatives 34 and 35 (Fig. 12).^96^ Of these esters only the carbonate-substituted compound 34 released the parent acid efficiently *in vivo*. 34 is purportedly hydrolysed at the carbonate group and then spontaneously disintegrates to risedronate in the bloodstream, but no evidence was given in support of this mechanism of activation. Presumably compounds 35 are more stable to degradation because their benzene rings are less electron rich. Prodrug 34 was administered to both fasted and fed rats *via* intraduodenal dosing (to mimic oral dosing with enteric-coated drug), after which more risedronate was recovered in the urine than with equimolar dosing of risedronate itself. This proves that cyclic acetal promoieties can be used to increase the gastrointestinal absorption of this bisphosphonate.

### Acyloxymethyl or alkoxycarbonyloxymethyl promoieties

**Scheme 2:**
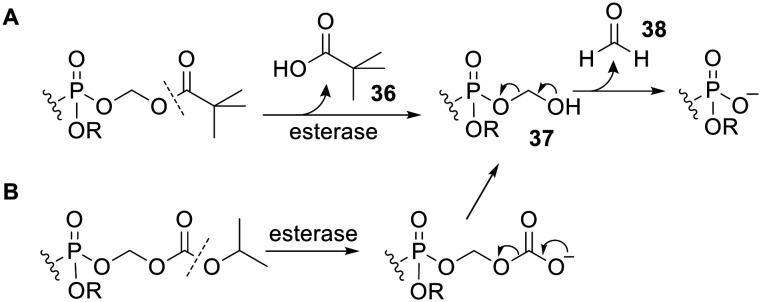
Mechanism of activation of (A) POM and (B) POC ester prodrugs.

Simple bisphosphonate esters are most likely to be hydrolysed by phosphodiesterases or phosphatases. However there are other types of esterases which can be used to activate prodrugs. For example, pivaloyloxymethyl (POM, pivoxil) and isopropoxycarbonyloxymethyl (POC, isoproxil) groups have been used to mask bisphosphonates.^97–99^ Release of the bioactive compound from both of these types of prodrug is triggered by (carboxyl)esterase-mediated hydrolysis (Scheme 2). This yields an unstable hydroxymethyl phosphonate 37 (*via* carbonate decarboxylation in the case of POC groups), which undergoes spontaneous fragmentation with expulsion of a phosphonate anion.^100^ It should be noted that the by-product of this reaction, formaldehyde 38, is toxic and carcinogenic.^101^ Furthermore, POM ester hydrolysis leads to intracellular release of pivalic acid 36, which is known to reduce carnitine levels as a result of excretion of pivaloyl carnitine into the urine.^102^ Nevertheless, adefovir dipivoxil and tenofovir disoproxil fumarate are FDA-approved prodrugs of (mono)phosphonate nucleosides^103^ (see below). In addition, other approved drugs, such as β-lactams pivampicillin, pivmecillinam and cefditoren pivoxil, also have the POM promoiety. Thus prodrugs using POM or POC promoieties remain viable options provided the effective dose of the prodrug is comparable to or less than that of these approved drugs and the treatment is not prolonged.

**Fig. 13:**
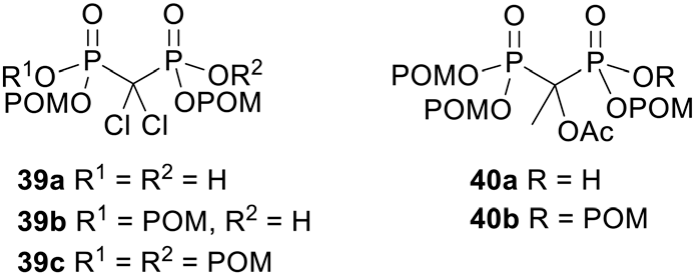
POM esters of clodronate and etidronate acetate studied by Vepsäläinen and coworkers.^89,104^

Vepsäläinen and coworkers were the first to test whether acyloxymethyl esters of bisphosphonates could be used as prodrugs.^104^ They synthesised *P*,*P*′-di-, tri- and tetra-POM esters of clodronate 39 (Fig. 13) and measured their log *P*_app_ values to be −2.1, 1.6 and 7.4 respectively. Hence the triester 39b possessed the most suitable lipophilicity for efficient oral absorption. The aqueous solubility of the tetraester 39c was too poor for its hydrolysis kinetics to be determined but the stabilities of the di- and tri-POM esters 39a and 39b were evaluated in pH 5.0 and pH 7.4 phosphate buffer, in 80% human serum and in 10% liver homogenate. In all media the triester 39b was more susceptible to degradation than the diester 39a. This can be rationalised by noting that each phosphonate in 39a is negatively charged and thus repels nucleophiles and is a poor leaving group, whereas in 39b the doubly masked phosphonate is electrically neutral. In phosphate buffer and human serum the half-life of 39b was greater than 1 hour whilst 39a was not degraded, but in rabbit liver homogenate both compounds were hydrolysed, with half-lives of 1.1 min for 39b and 14 min for 39a, eventually releasing clodronate quantitatively. Therefore tri-POM clodronate 39b has the required properties for a prodrug since it is adequately lipophilic, it is relatively stable in aqueous solutions and it releases the parent drug rapidly in the presence of liver enzymes. The enzymes responsible for removing the progroups were not elucidated, although the prodrugs were not hydrolysed in solutions containing either carboxylesterase (EC 3.1.1.1) or phosphodiesterase I (EC 3.1.4.1).

In another paper from the same group, tri-POM and tetra-POM esters of acetylated etidronate 40 (Fig. 13) were synthesised.^89^ The C–OH needed to be acylated otherwise a rearrangement to a P-C–O–P structure occurred. Again, hydrolysis of the tetraester 40b (log *P* ∼ 7.4) could not be studied due to poor aqueous solubility, whilst the triester 40a (log *P* = 0.6) was relatively stable in pH 7.4 and pH 5.0 phosphate buffer and in human serum (half-lives of 6.8 days, 10.5 days and 4.8 h respectively) and no etidronate was formed. In 10% rabbit liver homogenate, however, disappearance of 40a was much faster (half-life of 2.7 min) and etidronate 27 (Fig. 9) was eventually released quantitatively (55 h).

**Fig. 14:**
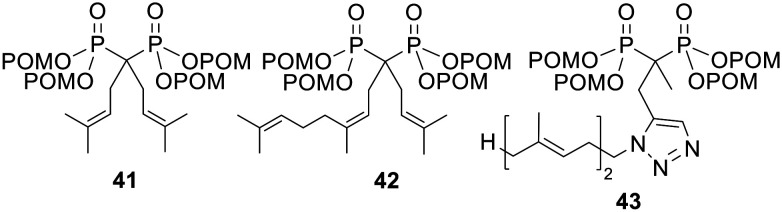
POM esters studied by Wiemer and coworkers.^105–107^

NBPs exert their cytotoxic effects through inhibition of FPPS (see above). Wiemer and coworkers found that isoprenoid-substituted bisphosphonates could also inhibit GGPPS and went on to synthesise tetra-POM derivatives (*e.g.*41–43, Fig. 14) in an attempt to improve cellular potency.^105–107^ This increased the clog *P* values of the most hydrophilic bisphosphonates by up to seven orders of magnitude (values for the acids were between 1.7 and 5.4, whilst values for the esters were between 8.9 and 11).^105^ Pivoxil modification also increased the cytotoxicity of the compounds towards K562 chronic myelogenous leukaemia cells – whereas the GI_50_ values were greater than 100 μM for all but one of the parent bisphosphonates, eight of the prodrugs had GI_50_ values of less than 10 μM. Furthermore, many of the POM esters were better able to inhibit Rap1a and Ras geranylgeranylation in K562 cells. Compound 42 in particular, displayed IC_50_ values of 1 μM, whilst its parent acid showed a value 25-fold higher. Compound 43 also had an approximately 10-fold greater potency than the corresponding bisphosphonate.^107^ Although no direct evidence was given for bisphosphonate release by prodrug hydrolysis, POM esters of three of the most active bisphosphonates were shown not to be able inhibit GGPPS.^105^

**Fig. 15:**
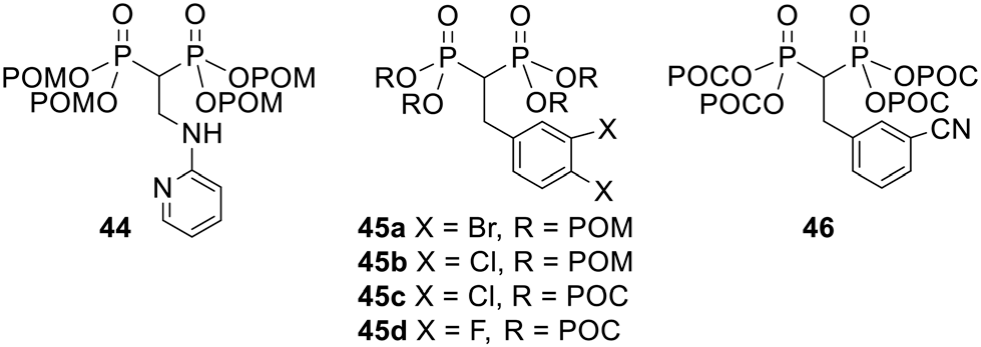
POM and POC esters studied by Zhang *et al.*^108^

POM/POC esterification also dramatically increased the cell growth inhibitory activity of bisphosphonates against a variety of cancer cell lines in an *in vitro* study by Zhang *et al.*^108^ For example, the tetra-POM ester 44 (Fig. 15, IC_50_ = 6.8 μM average over three different cell lines) was 20 times more potent than its corresponding acid (IC_50_ = 145 μM). Tetra-POM ester 45a (IC_50_ = 500 nM) was over 800-fold more potent than its acid form (IC_50_ = 442 μM) and tetra-POC ester 46 was active (IC_50_ = 29.7 μM) whereas its acid equivalent was not (IC_50_ > 1000 μM). Esters 45b–d were also active at low concentrations (IC_50_ = 1.49, 3.87 and 21.2 μM respectively) although the corresponding acids were not evaluated. Notably, the POM-protected prodrug 45b was active at lower concentrations than the POC equivalent 45c.

**Fig. 16:**
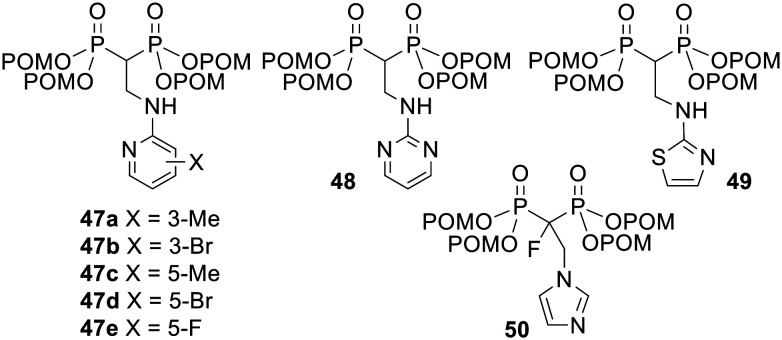
POM esters studied by Tanaka and coworkers.^109–111^

Similar conclusions were drawn by Tanaka and coworkers, *i.e.* POM esterification consistently increased growth inhibitory activities in U937 histiocytic lymphoma and EJ-1 bladder carcinoma cell lines.^109^ In addition the most potent compound 49 (Fig. 16, 0.023 ≤ IC_50_ ≤ 1.2 μM) was shown to be between 28- and 3700-fold more active than its unprotected version (3.7 ≤ IC_50_ ≤ 210 μM) in 10 solid tumour-derived cell lines and in 11 haemopoietic cell lines. Tanaka *et al.* went on to show that when Vγ2Vδ2 T cells were exposed to tumour cells incubated with POM-modified prodrugs 47–49 or their parent acids, the prodrugs stimulated the T cells to secrete TNF-α at lower concentrations than the acids.^110^ For example, compound 49 was between 80 and 1900 times more potent than its acid form in 22 tumour cell lines. Similarly, pretreatment of tumour cells with 1 μM 50 induced their lysis by Vγ2Vδ2 T cells to the same extent as 1000 μM of the corresponding acid.^111^ Unfortunately compounds such as 49 are too hydrophobic to be dissolved in polar solvents including water and ethanol.^112^ This presents a significant barrier to their use in the clinic, *e.g.* as adjuvants for adaptive immunotherapy, but 49 could be solubilised using a cyclodextrin and was very effective at stimulating the expansion in numbers of Vγ2Vδ2 T cells *ex vivo*.

**Fig. 17:**
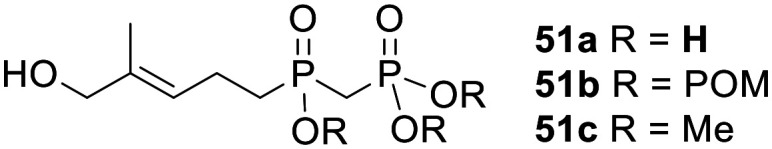
POM and methyl esters studied by Wiemer and coworkers.^113,114^

As with IPP and DMAPP (see above), (*E*)-4-hydroxy-3-methyl-but-2-enyl diphosphate (HMBPP), an intermediate in bacterial isoprenoid biosynthesis, is a natural ligand of the BTN3A1 receptor and induces γδ T cell proliferation *in vivo*.^76^ In studies intended to elucidate the mechanism of BTN activation Wiemer and coworkers tested a non-hydrolysable analogue 51a of HMBPP (a phosphinomethylphosphonate) and its tri-POM prodrug 51b (Fig. 17).^113,114^ A 72 hour exposure to the acid 51a stimulated Vγ2Vδ2 T cell proliferation from peripheral blood mononuclear cells with an EC_50_ of 26 μM, whilst the prodrug 51b displayed a 630-fold lower EC_50_ (0.041 μM).^113^ A trimethyl protected analogue 51c was also evaluated and, as expected, was found to be inactive (at concentrations up to 100 μM). Furthermore, after a 2 h exposure the tri-POM prodrug 51b was able to induce T cell-mediated lysis of K562 cells at a 150-fold lower concentration than 51a (EC_50_ values 0.28 and 41 μM respectively).

### Acyl promoieties

**Fig. 18:**
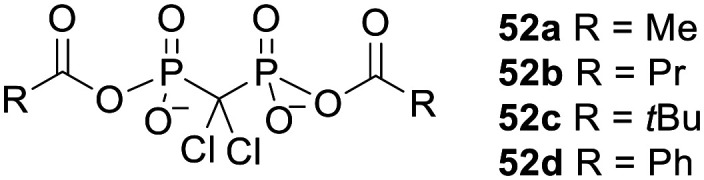
Clodronate anhydrides studied by Ahlmark *et al.*^115^

Bisphosphonate prodrugs can alternatively be produced by methods other than esterification. For example clodronate dianhydrides 52 (Fig. 18), in which each phosphonate is masked by a single acyl moiety, were synthesised by Ahlmark *et al.*^115–117^ These compounds were shown to be less soluble than clodronate in aqueous media, but unlike clodronate they maintained their solubility in the presence of Ca^2+^ ions.^115^ They were also found to be reasonably stable to hydrolysis in phosphate buffer at pH 7.4 (half-lives 15.2 h to 32.9 days) and pH 2.0 (half-lives 45 min to 11.9 days). Compounds 52c and 52d were most resistant to hydrolysis, which can be attributed to steric hindrance and resonance stabilisation respectively. On the other hand, in 80% human serum compounds 52a, 52b and 52d were extremely susceptible to enzymatic degradation, with all three compounds being completely hydrolysed after 1 min. 52c was presumably not such a good substrate for the hydrolytic enzymes in this medium (half-life 3.3 h) due to the bulkiness of the tertiary butyl group.

Vepsäläinen and coworkers found that it was not possible to synthesise bisphosphonate anhydrides in which more than two of the negative charges were masked with carboxylic acid progroups.^117^ The log *P* values of the clodronate dianhydrides were *ca.* −2.3 (ref. 115) and so although the prodrugs were substantially more lipophilic than the parent compound, they would likely still suffer from slow transcellular transport. Nevertheless, it is important to remember that lipophilicity is only one parameter that determines the effectiveness of a prodrug strategy. Indeed, compound 52c was able to liberate clodronate inside Caco-2 cells more efficiently than the more lipophilic tri-POM ester of clodronate 39b (Fig. 13, log *P* = 1.6) since it was hydrolysed faster in the intracellular environment.^118^

**Scheme 3:**
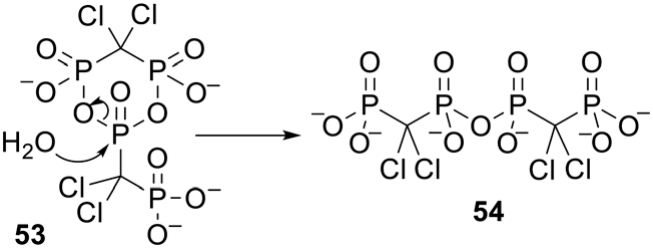
Partial hydrolysis of a cyclic dimer of clodronate studied by Vepsäläinen and coworkers.^119^

Interestingly, while trying to prepare clodronate anhydrides Vepsäläinen and coworkers discovered a cyclic clodronate dimer 53 (Scheme 3) that they envisaged could act as a ‘self-prodrug’ of clodronate.^119^ In fact the cyclic dimer was hydrolysed to a linear structure 54 in pH 7.4 phosphate buffer but this compound was stable to further degradation in the aqueous buffer and in human plasma.

### Nitrobenzyl with halobutylamine promoieties

**Scheme 4:**
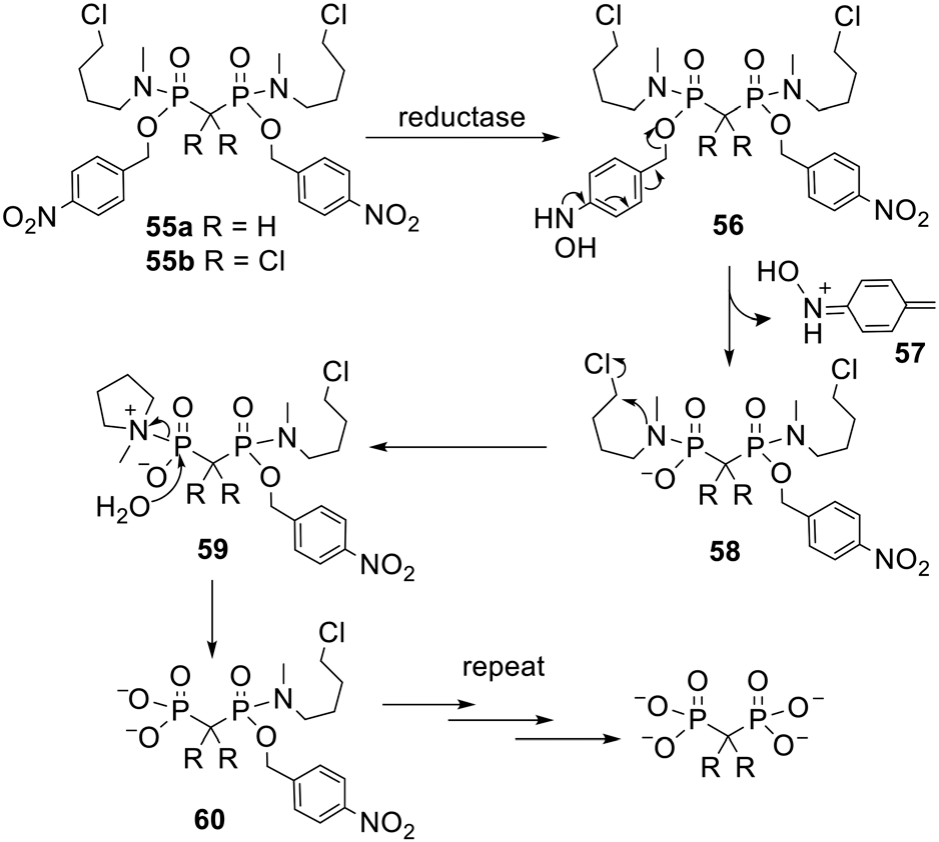
Mechanism of activation of bisphosphonamidate esters studied by Freel Meyers and coworkers.^121,122^

In prodrugs with multiple masking units removal of the first promoiety is often faster than removal of the second, which is faster than removal of the third and so on. For example, this has been shown to be the case for DiPPro NDPs^15,34,37^ and POM esters of clodronate.^104^ This phenomenon arises because successive removal of each progroup unmasks an extra negative charge in the molecule, which a) repels nucleophilic chemical species, b) reduces the leaving group ability of the anionic moiety and c) makes the compound a poorer substrate for hydrolytic enzymes such as esterases.^120^ Consequently Freel Meyers and coworkers designed new, efficiently activated bisphosphonate prodrugs 55 (Scheme 4) in which each phosphonate is masked with one halobutylamine and one nitrobenzyl group.^121^ In these bisphosphonamidate esters all the negative charges are concealed yet a single enzymatic activation step at each phosphonate moiety triggers a cascade of reactions that releases the fully unmasked parent molecule (Scheme 4). First, enzymatic reduction of the nitro group yields hydroxylamine 56. This then undergoes a spontaneous fragmentation in which the benzyl-phosphonate bond is cleaved. The nitrogen atom in the resulting phosphonamidate 58 is now electron rich and displaces chlorine, producing zwitterion 59 which is readily hydrolysed in aqueous media to intermediate 60, in which only one phosphonate moiety is masked. These steps are then repeated to release the parent bisphosphonate. This mechanism of activation is supported by the observation that methylene bisphosphonate (MBP) was released from 55a upon chemical reduction of the nitro group under model physiological conditions. One of the by-products of prodrug activation is electrophile 57, which may be toxic, so the safety of this type of prodrug would need to be checked.

Prodrug 55a was cell permeable and could release MBP inside A549 non-small cell lung cancer cells.^121^ As expected, treatment of intact cells with the prodrug afforded higher intracellular concentrations of the parent compound than treatment with comparable concentrations of the free bisphosphonate. Consistent with this improvement in cell uptake, clodronate prodrug 55b (IC_50_ = 4.4 μM) more potently inhibited the growth of A549 cells *in vitro* than clodronate itself (not active at 1 mM). In later work compound 55b was also found to have a more potent effect on cell growth and viability in SK-Mel-5 and UACC-62 melanoma cell lines.^122^ Moreover it was able to decrease tumour growth in a mouse xenograft model whereas no statistically significant effect was observed with equal doses of clodronate.

### Comparison with monophosph(on)ate prodrugs

There have been numerous reviews of monophosph(on)ate prodrugs, often focussing on the clinically approved prodrugs or those in clinical trials.^10–13,123–126^ As a result, only a brief overview will be given here.

**Fig. 19:**
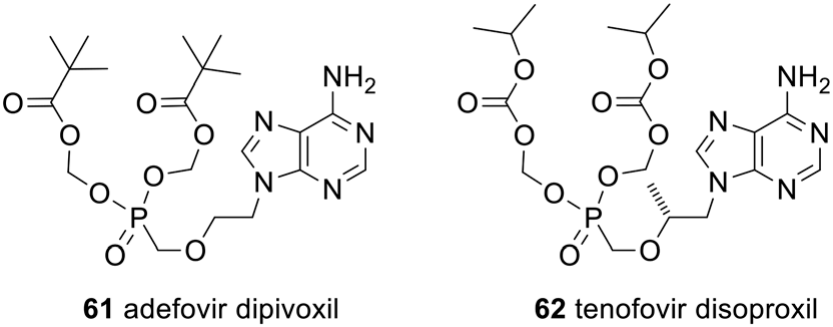
Clinically approved POM and POC prodrugs of nucleotide analogues.

Some of the strategies used are equivalent to those described above. For example, the clinically approved nucleotide analogue prodrugs adefovir dipivoxil 61 and tenofovir disoproxil fumarate 62 (Fig. 19) use POM and POC promoieties, respectively, to mask the phosphonate; see Scheme 2 for the mechanism of their activation. Both are used to treat hepatitis B but 61 (trade names Preveon and Hepsera) is used at a dose of 10 mg per day, whereas for 62 (brand name: Viread) the dose is 245 mg per day.^127^

However, most of the nucleotide analogue prodrugs that have entered clinical trials are based on phophoramidates. In these cases a phosphoramidase enzyme, Hint1, catalyses the final hydrolysis to give the nucleotide analogue. The natural substrate for this phosphoramidase is not clear but the Hint1 protein is also involved in a non-catalytic role in some signalling pathways and mutations in the *hint1* gene can cause neuropathy.^128^

**Scheme 5:**
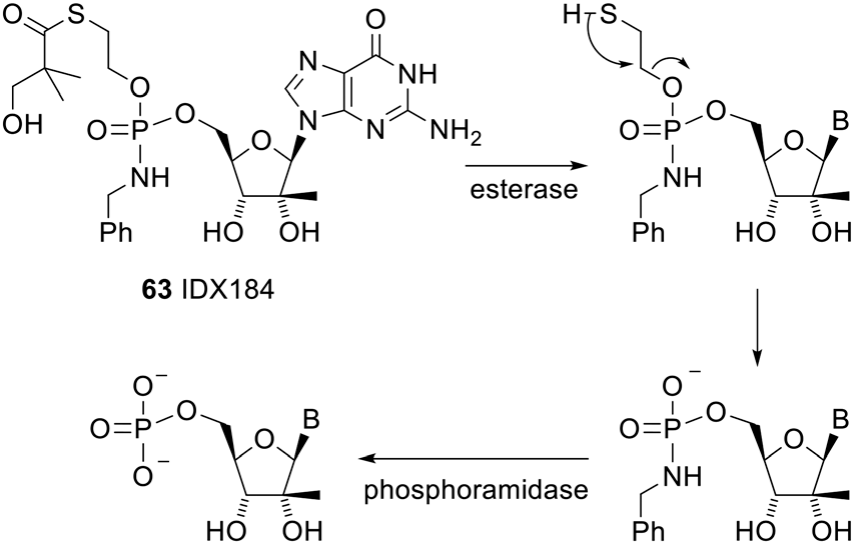
Mechanism of deprotection of the SATE prodrug IDX184.

One phosphoramidate prodrug that entered clinical trials, IDX184 63, used a *S*-acylthioethyl (SATE) group for the initial deprotection. The mechanism of deprotection of this prodrug is shown in Scheme 5.^126^

**Scheme 6:**
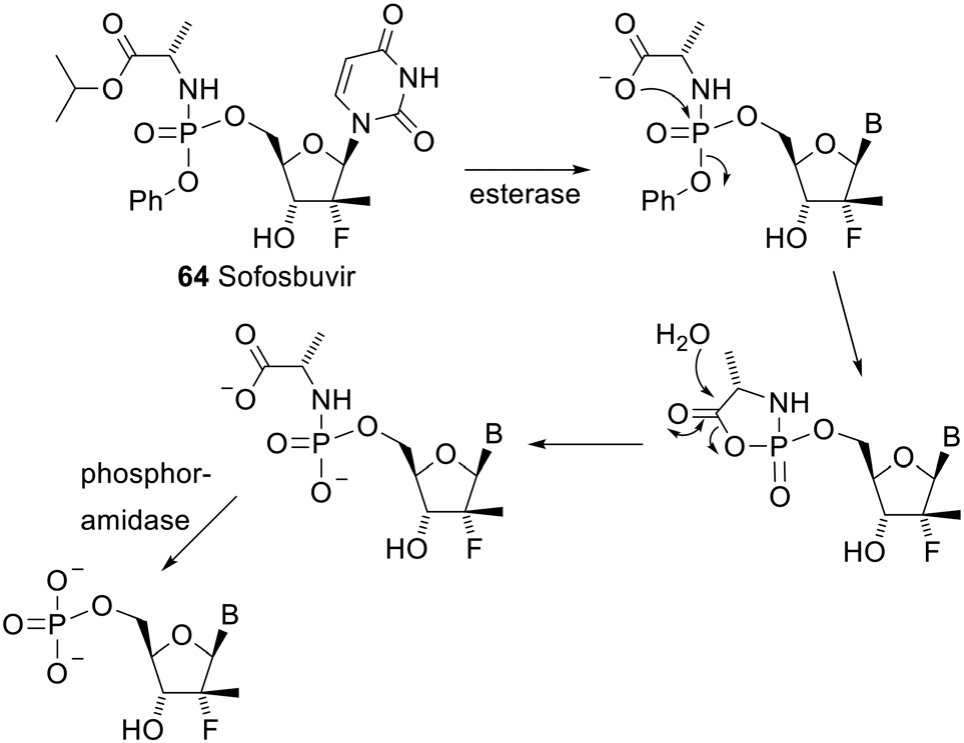
Mechanism of deprotection of the ProTide Sofosbuvir.

The two phosphoramidate prodrugs that have gained clinical approval both have an amino ester and a phenoxy group as the two phosphoryl substituents. This class of prodrug was developed by the McGuigan group at Cardiff and they are called ProTides. One of these approved drugs is sofosbuvir 64 (brand name Sovaldi). Its mechanism of deprotection is shown in Scheme 6.^126^ The esterase in the first step is predominantly cathepsin A or carboxylesterase 1. The other approved drug is tenofovir alafenamide (brand name Vemlidy), a second prodrug of tenofovir (see 62) with the same substituents on the phosphoryl group as sofosbuvir.

**Fig. 20:**
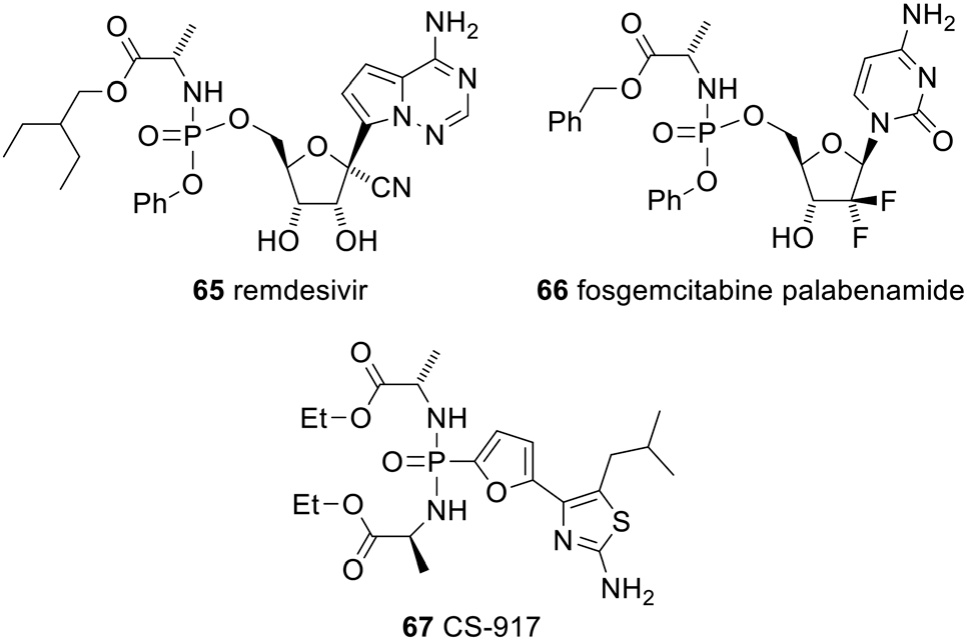
Various other clinically trialled phosph(on)ate prodrugs.

Another ProTide, remdesivir 65 (trade name Veklury), has been approved for emergency use in the Covid-19 pandemic in the USA and many other countries (Fig. 20). Many other ProTides have entered clinical trials. Most have been for the treatment of viral infections, but some have been for the treatment of other indications. For example, fosgemcitabine palabenamide 66 (brand name Acelarin) is in phase III clinical trials for the treatment of biliary tract cancer and was given Fast Track designation by the FDA in September 2021.^129^

Phospho-bisamidates can also be deprotected in a similar way to ProTides and CS-917 67 was entered into clinical trials for treatment of type 2 diabetes (the deprotected phosphonate is a potent inhibitor of fructose 1,6-bisphosphatase). However, ultimately it proved ineffective.^130^

It is interesting that the ProTide protection method has not been reported for pyrophosphates. Although the initial hydrolysis by an esterase should work, it may be that displacement of the phenoxy group fails because the nucleoside monophosphate is a better leaving group, or it may be that the final phosphoramidase-catalysed hydrolysis does not work on an amidate of a nucleoside diphosphate.

The 4-acyloxybenzyl esters used in the Di*PP*ro approach described above have also been used to protect monophosphates, and deprotection of the resulting esters in cells has been shown.^48,131–134^ However, these prodrugs have not featured in clinical trials. This may be because of concerns about toxicity of the quinonemethide by-product 12 released during hydrolysis (Scheme 1). Indeed, some 4-acyloxybenzyl derivatives have shown significantly increased toxicities relative to their parent monophosphates.^131,134^

All the monophosph(on)ate prodrugs discussed here require enzymic deprotection. Wiemer^135^ has pointed out that passive diffusion of the prodrug into and out of the cell is usually faster than the deprotection and, once deprotected, the phosph(on)ate is anionic and no longer able to diffuse out of the cell. Thus, the drug will accumulate within cells and it will reach a higher concentration in cells that have a higher activity of the required enzyme. This presents the possibility of targeting the drug to certain parts of the body where the deprotection occurs most rapidly. These principles will also apply to the pyrophosphate and bisphosphonate drugs discussed in this review.

## Conclusions and future directions

There is a clear need for efficient pyrophosphate prodrugs. However, the inherent lability of the phosphoric anhydride bond greatly complicates the design of such compounds. All attempts to create NDP prodrugs that are unmasked by chemical hydrolysis have been unsuccessful. On the other hand, Di*PP*ro compounds that are efficiently cleaved by intracellular (carboxyl)esterases have been shown to selectively release NDPs *in vitro* in T lymphocytes. It remains to be seen whether these prodrugs can be successful *in vivo*, as questions concerning their toxicity, cell specificity and stability in systemic circulation are yet to be addressed.

Bypassing the second phosphorylation step with an NDP analogue prodrug may well avoid a rate-limiting step in nucleoside analogue activation, as explained earlier. But this inevitably means that some other step becomes rate-limiting. One study^43^ on di*PP*ro prodrugs of ddU and d4U found that the third phosphorylation became rate-limiting, but there do not seem to be further studies on this aspect. The third phosphorylation is believed to be catalysed by NDP kinases that are relatively non-selective, with all nucleobases and NDPs as well as 2′-deoxyNDPs accepted.^17^ There are, however, a couple of nucleoside analogues that, when administered, are reported to accumulate the corresponding NDP analogue in cells, showing that the third phosphorylation is rate-limiting in these cases.^17^

Bisphosphonate prodrugs are also highly desired. In theory bisphosphonate prodrug design is less challenging as phosphonate groups (of which bisphosphonates are comprised) are generally stable to hydrolysis. Indeed, there are several examples of successful (mono)phosphonate prodrugs.^11,100^ Nevertheless there are still some challenging aspects of bisphosphonate prodrug development. For example: several bisphosphonates are UV-vis inactive and thus can only be detected (*e.g.* in biological media) after derivatisation.^121^ α-Hydroxybisphosphonates need hydroxyl group protection if all four phosphonate oxygens are to be substituted, or else they undergo a rapid isomerisation in which the P–C(OH)–P backbone rearranges to a P–C–O–P structure.^89,136^ Furthermore bisphosphonate prodrug synthesis can require selective derivatisation of four equally reactive functional groups.^16^ The most frequently reported type of bisphosphonate prodrug involves masking all four oxygens with pivaloyloxymethyl (POM) groups. However, the only bisphosphonate prodrugs that have so far been proven useful *in vivo* are those containing either phenyl, cyclic acetal or halobutylamine with nitrobenzyl promoieties. For further preclinical development of these prodrugs more data is now needed on their pharmacokinetic and pharmacodynamic properties including bioavailability, cell specificity and toxicity.

As pyrophosphate tetraesters are too prone to hydrolysis, whereas methylenebisphosphonate tetraesters are stable, it is surprising more use has not been made of bisphosphonate analogues of pyrophosphates in the prodrugs described so far. Prodrug 51b of HMBPP analogue 51a (Fig. 17) is the only example of which we are aware. We feel this is an avenue that may well prove fruitful.

Most of the pyrophosphate and bisphosphonate prodrugs that have been developed thus far are bipartite, consisting of a bioactive compound that is directly connected to a labile promoiety. The exceptions to this are the Di*PP*ro compounds and the bisphosphonate prodrugs containing either cyclic acetal or halobutylamine with nitrobenzyl alcohol promoieties. These are all tripartite prodrugs, in which a self-immolative linker separates the active compound from the portion of the progroup at which the initial activating reaction occurs. This (typically enzymatic) reaction triggers a rapid and spontaneous fragmentation by which the active compound is released. Use of a self-immolative linker usually improves the efficiency of activation, most likely by reducing the steric bulk and build-up of negative charge around the site of enzymatic processing. As detailed in this review, pyrophosphate and bisphosphonate prodrugs that are tripartite have generally been more successfully deprotected than bipartite examples. However, concerns remain about the toxicity of the by-product(s) derived from the linker and these need to be resolved before these become attractive drug candidates.

It is often challenging to develop artificial ligands that can compete with pyrophosphates for binding to macromolecules. On the one hand, the polyanionic motif of pyrophosphates and bisphosphonates allows high binding affinities to be achieved through ionic interactions, but on the other hand such a high charge density results in poor membrane permeability. Although this review has focused on the use of prodrug strategies to circumvent this issue, it is important to emphasise that other approaches exist. One option is to replace the pyrophosphate group with a bioisostere^137^ or a metal-binding group such as those used in some inhibitors of metalloenzymes including matrix metalloproteinase-9,^138^ angiotensin-converting enzyme^139^ and histone deacetylases.^140^ In addition, it may not even be necessary to find small molecules that can compete with the native ligand if allosteric sites can be targeted instead. Medicinal chemists can thus use a variety of strategies to achieve their aims.

## Author contributions

ESR did the majority of the literature research and wrote the first draft. AHYC and FJL advised, commented on drafts, made corrections, added short passages and checked the accuracy of the review.

## Conflicts of interest

There are no conflicts to declare.

## Supplementary Material
